# Prognostic Role of EGFR/p-EGFR in Patients With Nasopharyngeal Carcinoma: A Meta-Analysis

**DOI:** 10.3389/fonc.2021.697369

**Published:** 2021-08-19

**Authors:** Xishan Chen, Renba Liang, Lin Lai, Kaihua Chen, Xiaodong Zhu

**Affiliations:** ^1^Department of Oncology, The Fourth Affiliated Hospital of Guangxi Medical University, Liuzhou, China; ^2^Department of Oncology, Wuming Hospital of Guangxi Medical University, Nanning, China; ^3^Department of Radiation Oncology, Guangxi Medical University Cancer Hospital, Nanning, China

**Keywords:** EGFR, nasopharyngeal carcinoma, meta-analysis, prognosis, p-EGFR

## Abstract

**Background:**

The prognostic value of epidermal growth factor receptor (EGFR)/phosphorylated EGFR (p-EGFR) expression in nasopharyngeal carcinoma remains controversial. A meta-analysis was performed to investigate prognostic significance of EGFR/p-EGFR expression in patients with nasopharyngeal carcinoma.

**Methods:**

Literatures published before November 2020 were systematically searched in relevant databases, including PubMed, Web of Science, Embase, China National Knowledge Infrastructure (CNKI), and Wan fang databases. STATA 13 statistical software was used to analyze the pooled hazard ratio (HR) and 95% confidence interval (CI). Heterogeneity of the studies was examined by I^2^. Sensitivity and subgroup analysis were performed to explore sources of heterogeneity. The potential publication bias was assessed using both Egger’s and Begg’s tests.

**Results:**

A total of 20 literatures with 1545 patients were included for the meta-analysis. The meta-analysis results suggested that high expression of EGFR was significantly associated with poor overall survival (OS) (HR = 1.70, 95% CI: 1.24–3.15, P = 0.001) and disease-free survival (DFS) (HR = 2.58, 95% CI: 1.87–3.56, P = 0.000). However, it was not significantly associated with progression-free survival (PFS) (HR = 1.85, 95% CI: 0.90–3.82, P = 0.09) and distant metastasis-free survival (DMFS) (HR = 1.39, 95% CI: 0.73–2.67, P = 0.319). The subgroup analysis indicated that patients with EGFR high expression in studies of higher TNM stage (III–IV) ratio had significantly poor OS (HR = 2.27, 95% CI: 1.09–4.73, P = 0.03), but heterogeneity existed in studies (I^2^  =  95.1%, P = 0.000). Sensitivity analyses revealed that EGFR expression did not significantly affect OS by an individual study solely, indicating there was inherent heterogeneity in OS cohorts. There was no significant heterogeneity among eight studies in the DFS cohorts (I^2^ = 0%, P = 0.606). There was significant heterogeneity between EGFR expression and DMFS (I^2^ = 82.8%, P = 0.000). Sub-group analysis in differentiated carcinoma demonstrated a smaller heterogeneity (I^2^ = 33.2%). In addition, p-EGFR high expression had no significant correlation with OS (HR = 1.00, 95% CI: 0.88–1.14, P = 0.982) and DMFS (HR = 1.21, 95% CI: 0.96–1.52, P = 0.112). The heterogeneity among p-EGFR and OS studies was small (I^2^ = 21%, P = 0.26). There was no significant heterogeneity in the DMFS cohorts (I^2^ = 0%, P = 0.497).

**Conclusion:**

EGFR high-expression was significantly associated with poor OS and DFS, which may serve as a prognostic predictor for nasopharyngeal cancer.

**Systematic Review Registration:**

[https://www.crd.york.ac.uk/PROSPERO], identifier [number CRD42021258457].

## Introduction

Nasopharyngeal carcinoma (NPC) is a malignancy that arises from the epithelium of nasopharynx, having obvious regional characteristics and high incidence in China and southeast Asia (1). According to national cancer registry data in China, the incidence and mortality of NPC in Guangxi province rank first (2). Currently, the clinical TNM staging system is the principal prognostic indicator for NPC (3). However, clinical outcomes are different among patients with the same TNM stage (4). It seems that TNM stage alone is insufficient to predict individual clinical outcome. Several studies have shown that varied biological behavior and different prognosis was presented in the NPC patients with the same classification (5–7). Therefore, a reliable prognostic biomarker is necessary to improve individualized patient treatment and predict outcomes.

EGFR, belonging to the receptor tyrosine kinase family, plays an important role in regulation of proliferation and survival of tumor cells (8, 9). After ligand binding, EGFR is activated and forms homodimers or heterodimers, resulting in the phosphorylation and activation of multiple downstream signaling pathways, such as cellular differentiation, proliferation, and carcinogenesis (10, 11). Studies have demonstrated that EGFR is frequently overexpressed in NPC (12, 13). However, the relationship between EGFR expression and prognosis remains controversial. Several researches reported that high expression of EGFR was associated with poor prognosis (14–16), while other studies found no association between EGFR and prognostic value in NPC patients (17–19). Differences in study population’s characteristics and cutoff values may explain the discrepancies among different studies.

Phosphorylated-EGFR (p-EGFR) may be more predictive of patient outcome. Recent studies demonstrated that p-EGFR high-expression was associated with poorer prognosis in patients with sarcoma (20) and non-small cell lung cancer (21). In addition, some studies found that p-EGFR high-expression was closely related to nasopharyngeal cancer development (22, 23). Hence, we performed this updated meta-analysis to evaluate prognostic significance of EGFR/p-EGFR expression in patients with NPC.

## Materials and Methods

### Search Strategy

This meta-analysis was reported according to the Preferred Reporting Items for Systematic Reviews and Meta-Analyses (PRISMA) Statement and was registered at International Prospective Register of Systematic Reviews (number CRD42021258457). PubMed, Embase, Web of Science, CNKI, and Wan Fang Data were searched to identify relevant studies which were published before November 2020. The following words in English were used for retrieval of relevant studies: ((((((((EGFR) OR EGFR transcription factor) OR (epidermal growth factor receptor)) OR EGFR protein) OR pEGFR) OR phospho-EGFR) OR (phosphorylated signal epidermal growth factor receptor)) OR phosphorylated EGFR transcription factor) OR protein EGFR OR (erbB1)) OR (HER1) AND (((NPC) OR (nasopharyngeal carcinoma)) OR (nasopharyngeal neoplasm)) OR (nasopharyngeal cancer). In addition, the following words in Chinese were searched for relevant studies: nasopharyngeal cancer, EGFR, and phospho-EGFR.

### Inclusion Criteria

The following inclusion criteria were used in this study. (1) The tissue samples were from clinically diagnosed nasopharyngeal cancer patients. (2) Immunohistochemical (IHC) assay was performed to examine EGFR/p-EGFR expression. (3) HR and 95% CI was used to evaluate the association between EGFR/p-EGFR overexpression and survival time, or Kaplan-Meier (K-M) curves were used to estimate survival time. (4) When the results were reported in multiple publications, the most complete and recently reported data was extracted.

### Exclusion Criteria

The exclusion criteria were as follows: (1) recurrent or metastatic NPC tissue samples, (2) unable to obtain HR and 95% CI date or K-M curves or insufficient data, (3) the results collected from NPC cell lines or animal experiments, and (4) literatures published as letters, reviews, conference abstracts, case reports, or expert consensus.

### Data Collection

All articles were independently screened by the two investigators, and those studies not meeting the inclusion criteria were excluded. Any discrepancy was discussed and resolved by seeking opinions from a third party. The content of data extraction includes the following: (1) general information: first author, publication year, country, or region; (2) basic characteristics of studies: types of researches, number of patients, study size, patients’ mean age, follow-up time, detection method, ICH cutoff value, histological differentiation, TNM stage (I–II *vs*. III–IV), etc.; (3) primary data: HR and 95% CI of survival outcomes, including overall survival (OS) and/or disease-free survival (DFS)/progression-free survival (PFS)/distant metastasis-free survival (DMFS). The HRs and its 95% CI were extracted from the text indirectly or calculated from the K-M survival curve using Engauge Digitizer (version 12.2.1).

### Quality Assessment

Quality assessment was performed by two investigators separately according to the method of Hayden et al. (24) and the Reporting Recommendations for Tumor Marker Prognostic Studies (REMARK) (25), as previously reported by Almangush et al. (26). A score ≥10 was considered to indicate high quality articles.

## Statistical Analysis

HRs with 95% CI were used to evaluate the correlation of EGFR/p-EGFR high expression with the survival time of NPC patients. Meta-analysis was performed using Stata software (version 13.0). Heterogeneity among studies was assessed with the Cochran Q test and I^2^ test. The fixed effects model was used if there was no heterogeneity among studies (P ≥0.1, I^2^ < 50% in heterogeneity test). Otherwise, it was considered to have significant heterogeneity (P<0.1, I^2^ ≥ 50% in heterogeneity test), the random effect model was used, and the source of heterogeneity was explored using subgroup analysis or sensitivity analysis. The potential publication bias was evaluated using both Egger’s and Begg’s tests, and P > 0.05 was considered to have no publication bias.

## Results

### Literature Search Results, Characteristics and Quality Assessment of Included Studies

**Figure 1:**
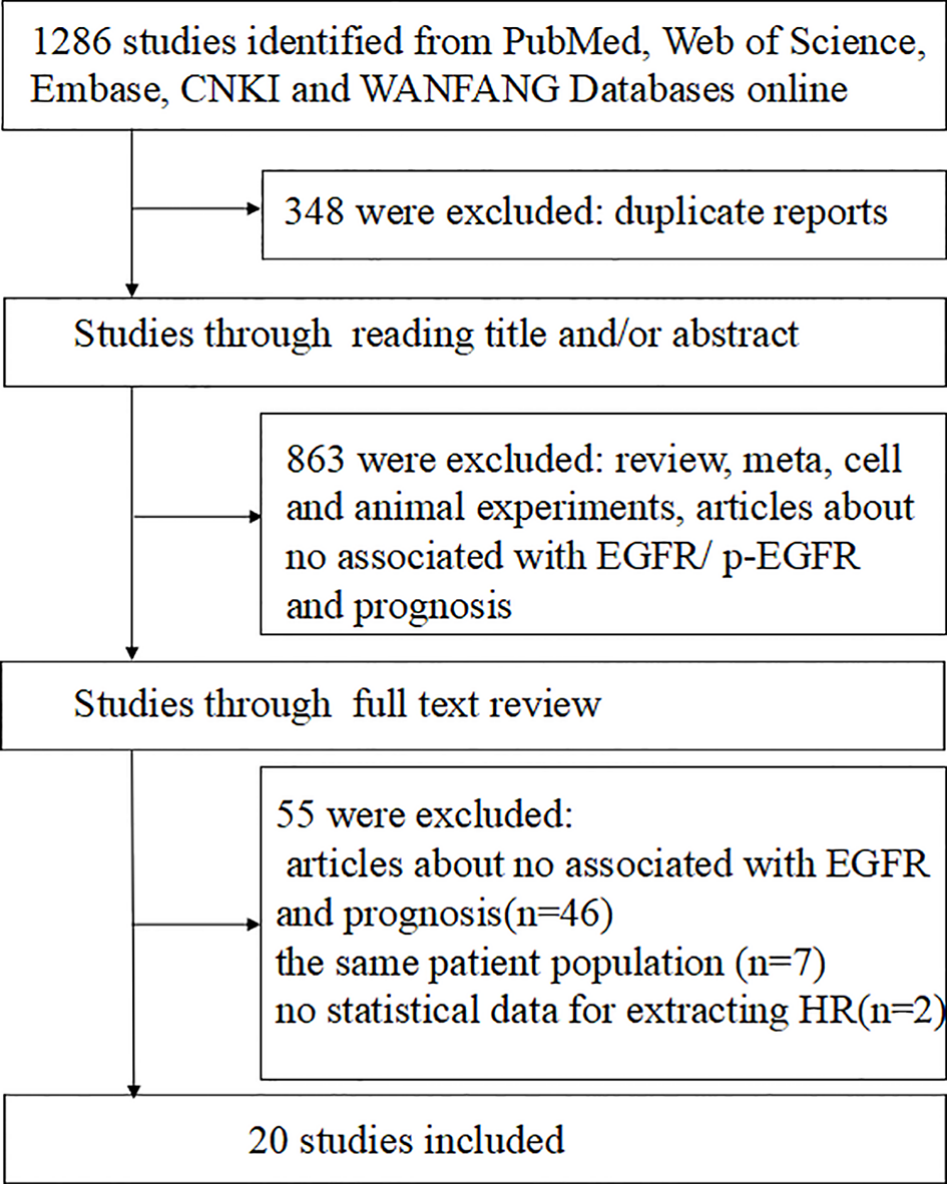
Flow chart of studies selection procedure.

A total of 1286 studies were identified, among which 680 articles were published in English and 606 in Chinese. After initial screening, 1211 studies were excluded, and 75 trials were retrieved for detailed assessment. After full-text screening, 20 studies with 1545 patients were eligible and included for our systematic review (12, 14–19, 22, 23, 27–35), of which three studies were published in Chinese and the others in English. These eligible studies were published from 2002 to 2019, and 19 of which were on EGFR and 3 on p-EGFR. The literature search flow is shown in **Figure 1**. The basic characteristics and quality assessment of the included studies are shown in **Tables 1** and **2**.

**Table 1:** Characteristics of included studies.

Author	Year	Type	Country	Study type	N	Age	Follo-up	Detection method	Histological differentiation (C *vs*. UC)	Clinical stage (I–II *vs*. III– IV)	Clinical outcome	EGFR effect	Treatment	Quality score
Fujii et al. (17)	2002	EGFR	Japan	RE	53	49	90.9m	IHC	45,8	24,29	DFS	NS	NACT+RT	9
Ma et al. (22)	2003	EGFR	China (Hong Kong)	PR	78	48	46m	IHC	0,78	29,49	OS	S	CCRT/RT	10
											DFS	S		
Chua et al. (27)	2004	EGFR	China (Hong Kong)	RE	54	NA	52m	IHC	0,54	23,31	DFS	S	NACT+	10
											DMFS	S	RT	
Leong et al. (28)	2004	EGFR	Singapore	PR	75	46	28.6m	IHC	0,75	26,49	OS	NS	NA	8
											DFS	NS		
Wang et al. (29)	2006	EGFR	China	RE	55	NA	NA	IHC	NA	7,48	OS	S	RT	7
Fang et al. (18)	2007	EGFR	China (Taiwan)	RE	30	17	NA	IHC	13,17	11,19	OS	NS	CCRT/RT+-AC	8
											DFS	NS		
Yuan et al. (23)	2008	EGFR	China	RE	110	47	65m	IHC	110,0	27,83	OS	NS	CCRT/RT+-NAC	7
											DMFS	NS		
	2008	p-EGFR	China	RE	110	47	65m	IHC	110,0	27,83	OS	NS	CCRT/RT+-NAC	7
											DMFS	S		
Yuan et al. (36)	2008	EGFR	China	RE	75	45	NA	IHC	75,0	24,51	OS	S	NA	8
Taheri-Kadkhoda et al. (14)	2009	EGFR	Sweden	RE	45	56	96m	IHC	NA	12,33	OS DFS	S	NACT+-AC+ERBT	10
											DMFS	S		
Huang et al. (12)	2010	EGFR	China (Taiwan)	RE	170	46	68m	IHC	76,94	71,99	OS	NS	CCRT/RT	10
											DMFS	NS		
	2010	p-EGFR	China (Taiwan)	RE	170	46	68m	IHC	76,94	71,99	OS	NS	CCRT/RT	10
											DMFS	NS		
Qi (33)	2010	EGFR	China	RE	55	45	60m	IHC	55,0	13,42	OS	NS	NACT+-CCRT/CCRT/RT	8
Kim et al. (30)	2010	EGFR	Korea	RE	38	48	30m	IHC	7,31	6,32	OS	NS	NA	10
											PFS	NS		
Kim et al. (19)	2010	EGFR	Korea	RE	69	50	54m	IHC	9,60	17,52	OS	NS	CCRT/ICRT/RT	10
Cao et al. (15)	2011	EGFR	China	RE	127	45	60m	IHC	NA	0,127	OS	S	IC+CCRT	8
											DFS	S		
Pan et al. (16)	2013	EGFR	China	RE	111	46	NA	IHC	NA	41,70	OS	S	CCRT/RT	9
											DFS	S		
											DMFS			
Zhang et al. (37)	2014	EGFR	China	RE	96	49	NA	IHC	NA	45,51	OS	S	CCRT/ICRT+-AC	9
Wu (34)	2015	p-EGFR	China	RE	107	50	31m	IHC	0,107	12,95	OS	NS	ICRT/CCRT	9
											PFS	NS		
Kang et al. (31)	2016	EGFR	Korea	RE	46	60	52m	IHC	NA	20,26	OS	NS	CCRT/RT	10
Mao et al. (32)	2019	EGFR	China	RE	31	44	NA	IHC	NA	3,28	OS	S	CCRT/ICRT+-AC, CTX	9
Wang et al. (35)	2019	EGFR	China	RE	120	55	43m	IHC	16,104	40,80	OS	S	CCRT/ICRT+-AC	8
											PFS	S		

RE, retrospective; PR, prospective; N, number of patients; NA, not available; S, significant (identifying EGFR/p-EGFR high-expression as a poor prognostic factor); NS, not significant; IRS, immunoreactive score; IC, induction chemotherapy; NACT, ICRT, induction chemotherapy followed by radiation Therapy; neoadjuvant chemotherapy; CCRT, concurrent chemoradiotherapy; AC, adjuvant chemotherapy; RT, radiotherapy; ERBT, external beam radiotherapy; CTX, cetuximab.

**Table 2:** Included studies were evaluated according to the REMARK guidelines.

Author (year)	Samples	Clinical data	Immunohistochemistry	Prognostication	Statistics	Classical Prognostic Factors
Fujii et al. (17)	A	A	A	I	I	A
Ma et al. (22)	A	A	A	A	I	A
Chua et al. (27)	A	A	A	I	A	I
Leong et al. (28)	A	A	A	A	I	I
Wang et al. (29)	I	A	A	I	I	I
Fang et al. (18)	A	A	A	A	A	A
Yuan et al. (23)	I	A	A	I	A	A
Yuan et al. (23)	I	A	A	I	I	I
Taheri-Kadkhoda et al. (14)	A	A	A	I	I	A
Huang et al. (12)	A	A	A	A	A	A
Qi (33)	I	A	A	A	A	I
Kim YJ et al. (30)	I	A	A	A	I	I
Kim TJ et al. (19)	I	A	A	A	A	A
Cao XJ et al. (15)	A	A	A	A	A	A
Pan et al. (16)	I	A	A	A	A	A
Zhang et al. (37)	A	A	A	I	I	I
Wu (34)	I	A	A	I	I	I
Kang et al. (31)	I	A	A	I	A	A
Mao et al. (32)	A	A	A	A	A	A
Wang et al. (35)	I	A	A	A	A	A

A, Adequate; I, Inadequate.

### Meta-Analysis Between EGFR/p-EGFR Expression and Prognosis

#### EGFR/p-EGFR Expression and OS

**Figure 2:**
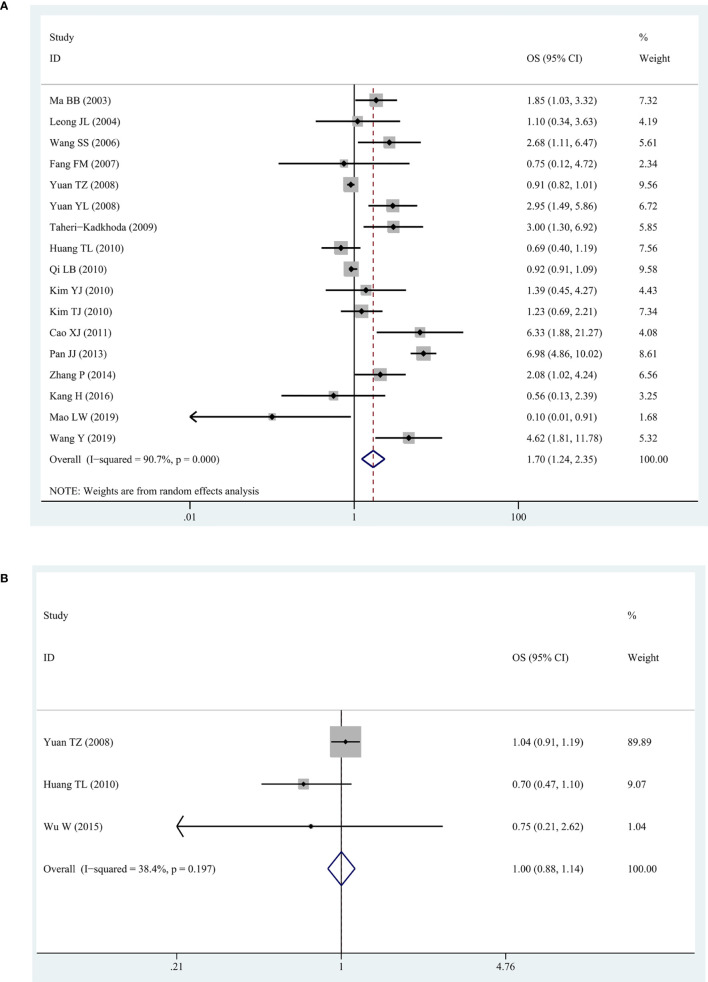
The forest map for relationship between EGFR/p-EGFR and OS in NPC. **(A)** EGFR and OS. **(B)** p-EGFR and OS.

We observed a high degree of heterogeneity among the 17 studies reporting EGFR and OS (I^2^ = 92%, P = 0. 006). Despite this, the pooled HR indicated a significantly shorter OS in patients with higher expression of EGFR (HR = 1.70, 95% CI: 1.24–2.35, P = 0.001) (**Figure 2A**). For all three studies about p-EGFR and OS, the pooled HR was 1.00 (95% CI: 0.88–1.14, P = 0.982), indicating that p-EGFR high-expression had no significant correlation with OS in patients with NPC (**Figure 2B**). In addition, there was no obvious heterogeneity between these studies (I^2^ = 38.4%, P = 0.197).

#### EGFR/p-EGFR Expression and DFS/PFS/DMFS

**Figure 3:**
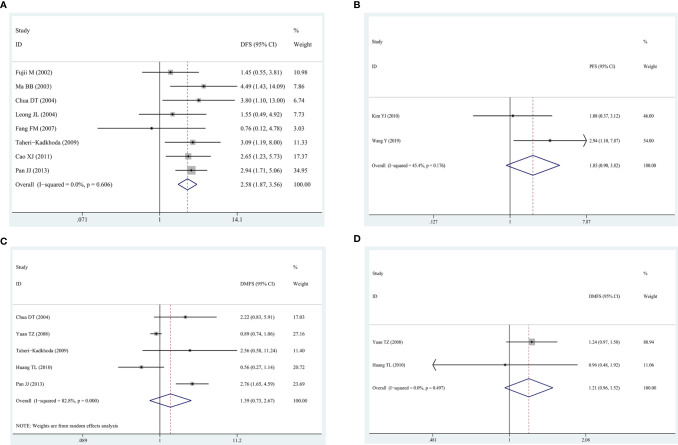
The forest map for relationship between EGFR/p-EGFR and DFS/PFS/DMFS in NPC. **(A)** EGFR and DFS. **(B)** EGFR and PFS. **(C)** EGFR and DFMS. **(D)** p-EGFR and DMFS.

Eight studies exploring the association between EGFR and DFS showed that EGFR high-expression was predictor of poorer DFS (HR = 2.58, 95% CI: 1.87–3.56, P = 0.000; I^2^ = 0%, P = 0.606) (**Figure 3A**), which was similar to the results of EGFR and OS. In two studies reporting EGFR and PFS, the pooled HR was 1.85 (95% CI: 0.90–3.82, P = 0.09), suggesting that patients with EGFR high-expression had a poor prognosis and there was an acceptable heterogeneity among studies (I^2^ = 45.4%, P = 0.176). In the five studies about DMFS, no significant association was found between DMFS and high-expression of EGFR with a pooled HR of 1.39 (95% CI:0.73–2.67, P = 0.319) (**Figure 3B**), but heterogeneity was significant among the studies (I^2^ = 82.8%, P = 0.000) (**Figure 3C**). On the other hand, in two studies reporting p-EGFR and DMFS, the pooled HR was 1.21 (95% CI: 0.96–1.52, P = 0.112) without heterogeneity (I^2^ = 0%, P = 0.497) (**Figure 3D**), revealing that high-expression of p-EGFR was not related to DMFS of patients with NPC.

### Subgroup and Sensitivity Analysis

**Figure 4:**
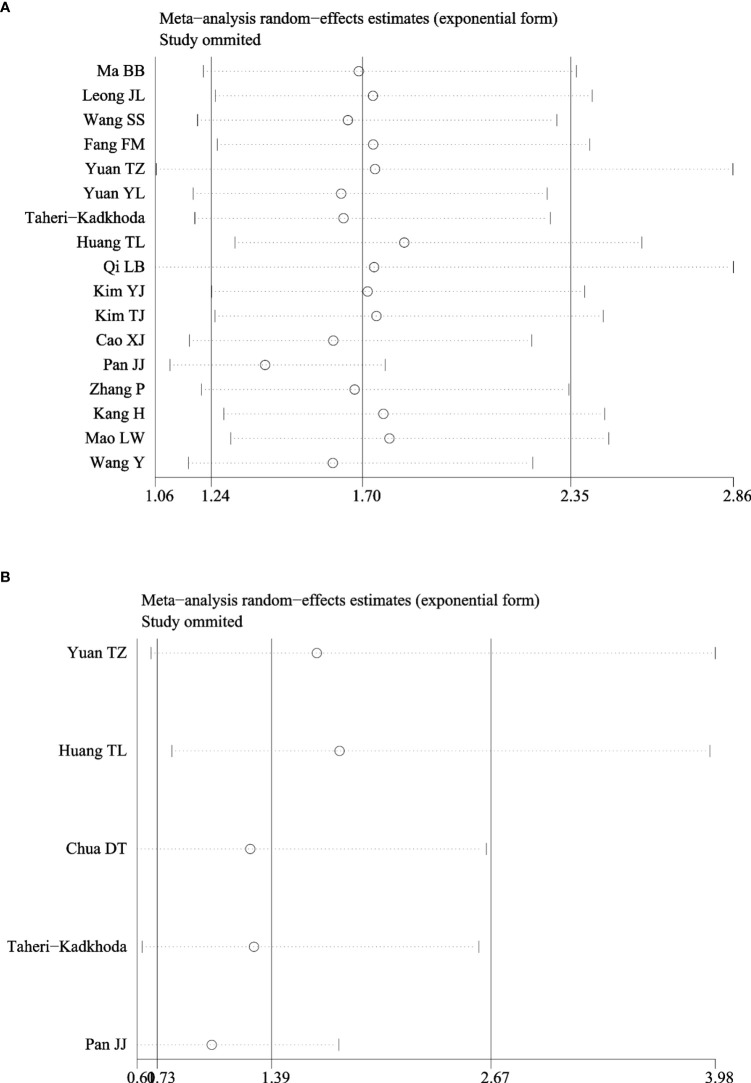
Sensitivity analysis of hazard ratios of EGFR for OS and DMFS. **(A)** EGFR and OS. **(B)** EGFR and DMFS.

As shown in **Table 3**, subgroup analyses showed that patients with EGFR high-expression in studies of higher TNM stage (III–IV) ratio divided using a median percentage of TNM stage I–II samples in entire samples had significantly poor OS (HR = 2.27, 95% CI: 1.09–4.73, P = 0.03). However, the heterogeneity still existed in those studies (I^2^ = 95.1%, P = 0.000). In addition, the prognostic value of EGFR was not significantly associated with the country, sample size, IHC cutoff value, and histological differentiation. Moreover, sensitivity analyses revealed that EGFR expression did not significantly affect OS by an individual study solely, indicating there was inherent heterogeneity in OS cohorts (**Figure 4A**). A subgroup analysis was performed for studies among EGFR and DMFS, finding that the heterogeneity obviously decreased in differentiated carcinoma subgroup (I^2^ = 33.2%) (**Figure 4B**).

**Table 3:** Subgroup analysis of relationship between EGFR and OS.

Marker	Survival outcome	N	Model	HR (95% CI)	P	Heterogeneity (I^2^, P)
EGFR	OS for Asian	16	R	1.65 (1.19–2.29)	0.003	91.0%, P = 0.000
EGFR	OS for higher rate in differentiated tumor	3	R	1.00 (0.81–1.23)	0.993	82%, P = 0.004
EGFR	OS for higher rate in undifferentiated tumor	7	R	1.38 (0.85–2.23)	0.189	57.4%, P = 0.029
EGFR	OS for cutoff 10%	7	R	1.53 (1.00–2.35)	0.052	95.1%, P = 0.000
EGFR	OS for cutoff 25%	5	R	2.04 (0.92–4.55)	0.081	78.4%, P = 0.001
EGFR	OS for higher TNM stage (I, II *vs*. III, IV)	8	R	2.27 (1.09–4.73)	0.03	95.1%, P = 0.000
EGFR	OS for lower TNM stage (I, II *vs*. III, IV)	9	R	1.29 (0.81–2.06)	0.289	65.4%, P = 0.003
EGFR	OS for number of samples (N > 100)	5	R	2.52 (0.84–7.54)	0.098	97%, P = 0.000
EGFR	OS for number of samples (N ≤ 100)	12	R	1.47 (1.00–2.16)	0.051	71.3%, P = 0.000

### Publication Bias

**Figure 5:**
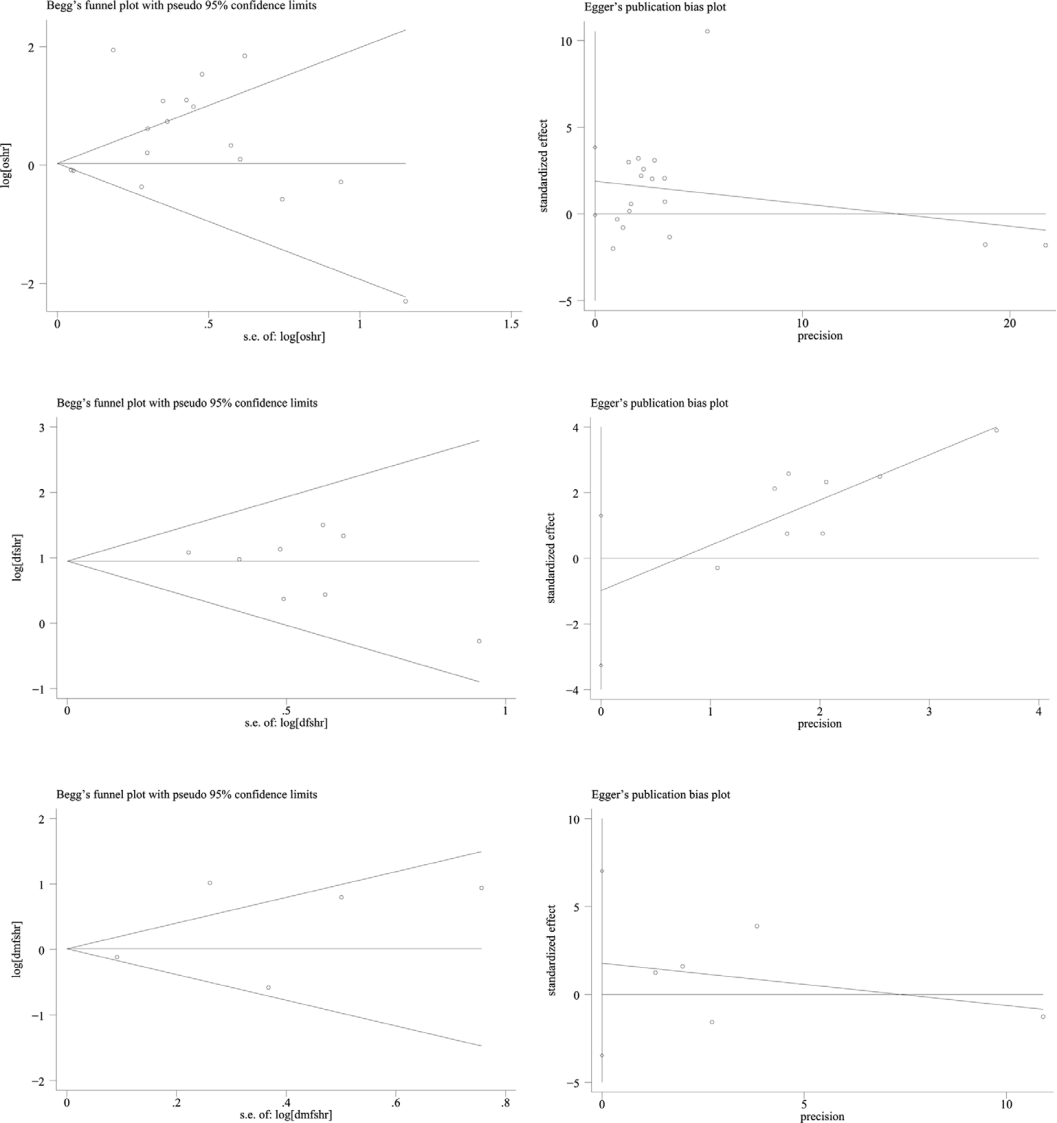
Publication bias funnel plot of EGFR and OS, DFS, DMFS: Begg’s test and Egger’s test. **(A)** EGFR and OS. **(B)** EGFR and DFS. **(C)** EGFR and DMFS.

Publication bias was evaluated using Begg’s test and Egger’s test. No significant publication bias was found among studies about EGFR and OS, DFS, and DMFS (all P-values were >0.05) (**Figure 5**).

## Discussion

EGFR high-expression and activation of downstream signaling pathways can promote cellular differentiation and contribute to aggressive tumor behaviors, such as increasing metastatic and migratory potential, chemotherapy and radiotherapy resistance, and stemness (38, 39). p-EGFR is an active form of EGFR and is crucial for EGFR signaling (40). It has been reported that p-EGFR was associated with poor prognosis of non-small cell lung cancer patients (21). Besides, patients with high expression of p-EGFR had shorter DMFS compared with those with low p-EGFR expression. However, the prognostic value of EGFR/p-EGFR expression in NPC remains controversial. Thus, the evaluation of relationship between EGFR/p-EGFR expression and prognosis may provide a more suitable strategy for individualized treatment of NPC.

Our meta-analysis showed EGFR could predict the outcome of patients with NPC. The pooled HRs for both OS and DFS indicate an important prognostic role for EGFR in NPC. Furthermore, the results of this meta-analysis are in accordance with the findings of previous meta-analysis (41, 42). However, the association between p-EGFR expression and the prognosis of NPC has not yet been assessed in the previous meta-analysis. In our meta-analysis, high-expression of p-EGFR was not significantly associated with OS (HR = 1.00, 95% CI: 0.88–1.14) and DMFS (HR = 1.21, 95% CI: 0.96–1.52). Additionally, heterogeneity testing displayed significant heterogeneity when analyzing OS and DMFS. Subgroup analyses revealed that patients with EGFR high expression in studies of higher TNM stage (III–IV) ratio had significantly poor OS, but heterogeneity existed in studies (I^2^ = 95.1%, P = 0.000). EGFR high-expression was not significantly associated with the country, sample size, IHC cutoff value, and histological differentiation. Sensitivity analyses also revealed that EGFR expression did not significantly affect OS by an individual study solely, indicating there was inherent heterogeneity in OS cohorts. In subgroup analysis with EGFR and DMFS, heterogeneity was reduced to I^2^ = 33.2% when we combined studies of differentiated carcinoma, indicating that the difference in tumor histology may be another source of heterogeneity and undifferentiated carcinoma was more likely to metastasize. In this study, no publication bias was observed according to both Begg’s test and Egger’s test in studies reporting OS, DFS, and DMFS, which proved the stability of our study.

Some of the included studies had deficiencies in some parameters according to the REMARKS guidelines, such as a potential ambiguity in the distinction between OS and disease specific survival in some of the included studies. There is no doubt that our study has serval limitations. Firstly, the studies included mainly focused on the patients in China, with insufficient data to examine the differences in trends by ethnic groups. Secondly, differences in quality of all included studies may affect the reliability of the results. Thirdly, the reliability and stability of the IHC results is related to the detection levels of research institutions and researchers themselves. Finally, we calculated the HR estimates from the K-M survival curves when some of the HRs with 95% CI were not directly extracted from the studies, which may be different from actual value.

In conclusion, EGFR high-expression is associated with shorter OS and DFS, suggesting that it may serve as a potential prognostic factor for patients with NPC. However, p-EGFR expression may not be used as a predictor of survival prognosis in patients with NPC, which needs to be confirmed in additional prospective, multicenter studies in the future.

## Data Availability Statement

The original contributions presented in the study are included in the article/supplementary material. Further inquiries can be directed to the corresponding author.

## Author Contributions

All authors listed have made a substantial, direct, and intellectual contribution to the work and approved it for publication.

## Funding

This work was supported by National Natural Science Foundation of China (81760544), the Key Research and Development Program Project of Guangxi Zhuang Autonomous Region (Grant No. GuikeAB18221007), the Independent Project of Key Laboratory of Early Prevention & Treatment for Regional High‐Incidence‐Tumor (Grant No. GKE2019-17), Guangxi Science and Nature Foundation Project (2017GXNSFBA198005), the Scientific Research & Technical Development Project of Wuming District, Nanning city (No. 20200214), Liuzhou City Science and technology research projects (2019AF10601), Liuzhou City Science and technology research projects (2018BJ10303), and Department of Health of Guangxi Zhuang Autonomous Region Self-Raised Funds Project (Z20200269 and 20200856). The Basic Ability Enhancement Program for Young and Middle-aged Teachers in Higher Education Institutions of Guangxi (No.2021KY0283, No. 2021KY0091) and Science Foundation for Distinguished Young Scholars of Guangxi University of Chinese Medicine (No. 2020JQ001).

## Conflict of Interest

The authors declare that the research was conducted in the absence of any commercial or financial relationships that could be construed as a potential conflict of interest.

## Publisher’s Note

All claims expressed in this article are solely those of the authors and do not necessarily represent those of their affiliated organizations, or those of the publisher, the editors and the reviewers. Any product that may be evaluated in this article, or claim that may be made by its manufacturer, is not guaranteed or endorsed by the publisher.
